# Computer-Aided Diagnosis of Melanoma Subtypes Using Reflectance Confocal Images

**DOI:** 10.3390/cancers15051428

**Published:** 2023-02-23

**Authors:** Ankita Mandal, Siddhaant Priyam, Hsien Herbert Chan, Bruna Melhoranse Gouveia, Pascale Guitera, Yang Song, Matthew Arthur Barrington Baker, Fatemeh Vafaee

**Affiliations:** 1 School of Biotechnology and Biomolecular Sciences, University of New South Wales (UNSW Sydney), Sydney 2052, Australia; 2 Department of Mechanical Engineering, Indian Institute of Technology (IIT Delhi), Delhi 110016, India; 3 Department of Electrical Engineering, Indian Institute of Technology (IIT Delhi), Delhi 110016, India; 4 Department of Dermatology, Princess Alexandra Hospital, Brisbane 4102, Australia; 5 Sydney Melanoma Diagnostic Centre, Royal Prince Alfred Hospital, Sydney 2006, Australia; 6 Melanoma Institute Australia, The University of Sydney, Sydney 2006, Australia; 7 School of Computer Science and Engineering, University of New South Wales (UNSW Sydney), Sydney 2052, Australia; 8 UNSW Data Science Hub, University of New South Wales (UNSW Sydney), Sydney 2052, Australia

**Keywords:** melanoma, Reflectance Confocal Images, machine learning, artificial intelligence

## Abstract

**Simple Summary:**

Melanoma is a serious public health concern that causes significant illness and death, especially among young adults in Australia and New Zealand. Reflectance confocal microscopy is a non-invasive imaging technique commonly used to differentiate between different types of melanomas, but it requires specialized expertise and equipment. In this study, we used machine learning to develop classifiers for classifying patient image stacks between two types of melanoma. Our approach achieved high accuracy, demonstrating the utility of computer-aided diagnosis to improve expertise and access to reflectance confocal imaging among the dermatology community.

**Abstract:**

Lentigo maligna (LM) is an early form of pre-invasive melanoma that predominantly affects sun-exposed areas such as the face. LM is highly treatable when identified early but has an ill-defined clinical border and a high rate of recurrence. Atypical intraepidermal melanocytic proliferation (AIMP), also known as atypical melanocytic hyperplasia (AMH), is a histological description that indicates melanocytic proliferation with uncertain malignant potential. Clinically and histologically, AIMP can be difficult to distinguish from LM, and indeed AIMP may, in some cases, progress to LM. The early diagnosis and distinction of LM from AIMP are important since LM requires a definitive treatment. Reflectance confocal microscopy (RCM) is an imaging technique often used to investigate these lesions non-invasively, without biopsy. However, RCM equipment is often not readily available, nor is the associated expertise for RCM image interpretation easy to find. Here, we implemented a machine learning classifier using popular convolutional neural network (CNN) architectures and demonstrated that it could correctly classify lesions between LM and AIMP on biopsy-confirmed RCM image stacks. We identified local z-projection (LZP) as a recent fast approach for projecting a 3D image into 2D while preserving information and achieved high-accuracy machine classification with minimal computational requirements.

## 1. Introduction

Reflectance confocal microscopy (RCM) is an in vivo imaging modality that enables large cutaneous lesions in cosmetically sensitive areas to be visualised to the depth of the papillary dermis without the requirement of a biopsy for formal histological assessment. The changes seen in Lentigo maligna (LM) and atypical intraepidermal melanocytic proliferation (AIMP, elsewhere known as atypical melanocytic hyperplasia, or AMH) involve the levels above the papillary dermis and are thus ideal candidates for the use of RCM for diagnosis [1,2].

Distinguishing between AIMP and LM is important because LM usually requires some form of definitive treatment before it may progress to invasion and the possibility of metastasis (lentigo maligna melanoma). AIMP, in contrast to LM, can continue to be monitored in vivo and tends not to respond to topical or radiotherapy treatments [2]. A number of clinical, histological, and RCM criteria have been proposed and validated to assist in distinguishing AIMP and LM: primarily non-edged papillae and round large pagetoid cells, and minor criteria: three or more atypical cells at the dermoepidermal junction in five RCM fields, and follicular localisation of atypical cells and nucleated cells within the dermal papillae. The presence of a broadened honeycomb is a significant negative feature for LM and is more suggestive of a benign seborrheic keratosis [3]. Nevertheless, it can be difficult to distinguish early LM from AIMP, given the common histological features of basal atypical melanocytic hyperplasia [4]. Further complicating the issue, AIMP has been shown to be, in fact, LM on further excision in 5% of cases [5]. Predictors of AIMP progression to LM have not been well defined, though they could include a target-like pattern and a high-density vascular network on dermoscopy and the presence of contact between dendritic cells on RCM [2].

RCM enables the longitudinal study of large heterogeneous lesions, with non-invasive and spatiotemporal tracking of heterogeneity. Computer-aided diagnosis can help to address the issue of access to diagnostics since the diagnosis and image acquisition can be physically separated (through remote acquisition), and computer-aided or entirely computational diagnosis can allow far greater patient throughput. However, a gold standard for borderline or uncertain malignancy does not exist, and current criteria are neither reproducible nor accurate [6]. Machine learning (ML) approaches can also be used to predict prognosis and have been employed in prostate and breast cancer to determine grades of differentiation that hold clearly defined risks of progression and prognostic outcomes [7,8].

Thus far, the use of ML on RCM datasets has been hampered by the limited availability of RCM infrastructure and labelled datasets in comparison to the extensive public libraries used to achieve dermatologist-level performance on clinical/dermoscopic images [9]. Nonetheless, a few successful applications of machine learning to RCM data exist: ML systems have been employed in the diagnosis of BCC [10] and in the diagnosis of congenital pigmented macules in infants [11]. Deep neural networks have also been employed in RCM image quality assessment, assisting the interpretation of RCM mosaics and automated detection of cellular and architectural structures within the skin [12,13,14,15,16]. RCM images have a cellular resolution at a range of depths and can be recorded as image stacks that can be reconstructed into a three-dimensional (3D) volume for an associated tissue. Generally, the classification of such 3D volumes is less established than the classification of two-dimensional (2D) images with existing computer vision approaches, and processing of 3D volumes is computationally more expensive than 2D image analysis [17].

Here, our hypothesis was that the projection of 3D virtual stacks into single 2D images could deliver high-accuracy machine binary classification between LM and AIMP lesions with reduced computational requirements and improved predictive performance, particularly in cases where sample sizes are limited. Our aim was to demonstrate high-accuracy machine classification of LM and AIMP lesions, utilising projections of RCM stacks that had been validated by clinician diagnosis and biopsy. In the following sections, we outline the design strategy employed in this study involving the use of multiple popular pre-trained convolutional neural network (CNN) architectures as well as a custom-made lightweight CNN model. Additionally, we combined CNN-based feature extractions with traditional machine learning (ML) classifiers. To ensure the validity of our results, we implemented strategies to reduce sample imbalance, mitigate the risk of overfitting, and enhance model robustness. We then consider in more detail specific example outcomes to examine which features are used for classification, what properties of image stacks may lead to misclassification, and the role of projection in our classification pipeline and its limitations. We identified the local z-projection (LZP) as a recent fast approach for projecting a 3D image into 2D while preserving information [18] and implemented our classifier on minimal computational architectures to achieve high accuracy.

## 2. Methods

### 2.1. Study Design and Participants

The study population comprised a total of 110 patients who attended the Sydney Melanoma Diagnostic Centre (Royal Prince Alfred Hospital, NSW) and the Melanoma Institute Australia RCM clinic between January 2019 and December 2020 who had biopsy-proven LM or AIMP lesions. Of note, the pathology department of these two units is a tertiary centre where expert dermatopathologists review all borderline cases to establish a consensus. A total of 517 RCM stacks were obtained for these patients from the RCM image database (HREC/11/RPAH/123—X15-0392 Sydney Local Health District Ethics Review Committee (RPAH zone)).

### 2.2. RCM Acquisition Procedure and Exclusion Procedure

Atypical, pigmented lesions were clinically identified as fulfilling the criteria for atypical change and scanned using a handheld Vivascope 3000 (Vivascope, Munich, Germany). Areas representing the diagnosis were identified by a trained confocal specialist, and stacks of 28–40 images (750 × 750 µm field of view with 3.5–5.0 µm depth spacing) were collected from patients at each site. Stacks were excluded when they were targeted at the margins of the lesions. Following imaging, areas with RCM-detected atypia were biopsied, and pathology was confirmed via formal histological diagnosis to create our ground truth. For slice-level classification, the clinician revisited each stack and for each individual image in the stack assigned a diagnosis of LM, AIMP, or neither.

### 2.3. Image Processing

**Figure 1:**
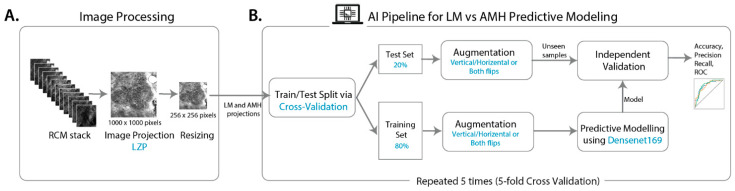
The schematic workflow of the study comprising image processing including projection and resizing (**A**), and deep learning model development and validation (**B**).

Individual images were exported from microscope software Vivascan (Vivascope ID) as 24-bit TIFF single images according to z-slice. Folders of individual TIFFs were imported into FIJI (ImageJ reference) as a virtual stack, and then initial projections were calculated using z-projection with the maximum and median. For subsequent classification using predictive modelling, stacks were projected using the FIJI plugin for LZP (https://biii.eu/local-z-projector), an optimal method for structure-specific projections that can be computed rapidly [18]. LZP was run in default settings for these stacks for the reference surface, with a max of the mean method with a 21-pixel neighbourhood search size and a 41-pixel median post-filter size and using maximum intensity projection (MIP) to extract the projection. Projections were then exported as 8-bit JPGs (1000 × 1000 pixels) and uploaded to Google Drive, where they were read using cv2.imread [19] and resized to 256 × 256 pixel images. Augmentation was performed on the AIMP data set using cv2 similarly (8 images augmented to 32 images by adding either horizontal flip or vertical flip or both horizontal and vertical flips). Resizing to 256 × 256 pixels was carried out using the cv2 resize function with inter-cubic interpolation. For the slice-level ternary classification, individual TIFFs were read in using cv2.imread and resized to 256 × 256-pixel images. Figure 1A illustrates the schematic workflow of image processing (projection and resizing).

### 2.4. Predictive Modelling

#### 2.4.1. Model Development

Different popular CNN architectures were employed to classify AIMP vs. LM projections, including ResNet50 [20], ResNet101 [21], InceptionV3 [22], VGG16 [23], and DensNet169 [24]. These models were pre-trained on ImageNet [25], and the model parameters were fine-tuned on RCM projections. We also developed a 6-layer CNN to evaluate the predictive performance on a simple architecture that is potentially less prone to overfitting. The Adam optimisation algorithm [26] was adopted to optimise the learning rate of neural network parameters for all the architectures except for ResNet50 and InceptionV3, for which the RMSProp algorithm [27] was used. Images were augmented to increase sample sizes. The strategy used for augmentation was flipping (vertical, horizontal, and a combination of both). To extend the diversity of the models evaluated, we also combined deep-learning-based feature extraction with other traditional classifiers. Accordingly, latent features were extracted from the DenseNet169 and ResNet50 models (i.e., the first and second best-performing CNN models). Extracted latent features derived from ResNet50 have shown better performance once used as predictive variables of different commonly-used classifiers, including support vector machines (SVM), random forest (RF), and k-nearest neighbours (KNN), and AdaBoost [28], using default hyperparameters (as detailed in Supplementary Table S1). All models were developed in Python using Keras neural network library on the TensorFlow platform.

#### 2.4.2. Model Validation and Performance Metrics

The *k*-fold cross-validation [29] was employed for model validation to give a more robust and generalisable estimate of the model’s predictive performance. Accordingly, patients (not images) were split into test and train sets. The test set was held out, and the training set was randomly partitioned into *k* complementary subsets; one is taken as a validation set for model optimisation and the rest as the training set. Projected images were randomly split into test and train sets with a constraint that multiple projected stacks from a single patient were included in either test or train sets (i.e., patient-level splitting) to avoid any potential information leakage from train to test set. Accordingly, roughly 20% of projections were withheld as a test set. This process was repeated *k* times so that each subset would be considered as a validation set in one iteration. The performance metrics over the holdout test set were then evaluated and reported for each of the *k* models trained. We performed a 5-fold cross-validation, and in each iteration, we used multiple metrics to measure the prediction performance on the test set, including accuracy (rate of correct classifications), recall or sensitivity (true positive rate), precision (positive predictive value), and F1-score, that is the harmonic mean of the precision and recall, i.e., F1-score = 2/(recall^−1^ + precision^−1^). The quality of models was also depicted by the receiver operating characteristic (ROC) curve, which plots the true positive rate (i.e., sensitivity) against the false positive rate (i.e., 1-specificity) at various threshold settings [30]. The area under the ROC curve (AUC) was computed, which varies between 0.5 and 1. The higher the AUC, the better the performance of the model at distinguishing between AIMP versus LM; a random or uninformative classifier yields AUC = 0.5. The confusion matrix was also reported on the selected model detailing the total number of correct and incorrect predictions, i.e., true positives (TP), false positives (FP), true negatives (TN), and false negatives (FN). For a sensible model, the diagonal element values will be high (TP and TN), and the off-diagonal element values will be low (FP and FN). The workflow diagram for developing and validating a deep learning model is presented in Figure 1B, highlighting the key steps involved in the process.

### 2.5. Prediction Interpretation

We used the Gradient-weighted Class Activation Mapping (Grad-CAM) [2,31] algorithm to produce visual explanation heatmaps highlighting the important regions in the images that contribute to the decision made by the best-performing CNN model (i.e., DenseNet169). Accordingly, AIMP and LM projected images in the test sets were run through the DenseNet169 model that is cut off at the layer for which we want to create a Grad-CAM heatmap. The layer output and the loss were then taken, and the gradient of the output of the model layer with respect to the model loss was found. The gradient which contributes to the prediction was taken, reduced, resized, and rescaled so that the heatmap can be overlaid with the original image.

### 2.6. Statistical Analysis

The statistical hypothesis tests comparing the significance of the performance enhancement comparing the best performing method (DenseNet169) and other competing algorithms were conducted using the paired two-tailed *t*-test. Statistical significance was defined as a *p*-value < 0.05. Statistical analyses were performed in R using the ‘stats’ library.

## 3. Results

### 3.1. Benchmarking of CNN Architectures through Classification Performance

Overall, 517 RCM stacks of 28–40 images (750–750 µm with 3.5–5.0 µm depth spacing) were collected from 110 patients (Supplementary Table S2). Figure 1 illustrates the image processing and diagnostic modelling pipeline developed in this study. The imbalance in the proportion of LM versus AIMP cases was partially handled by augmenting AIMP images by flipping them horizontally, vertically, and in both directions. Together, the training set included 537 projections (389 labelled LM and 148 AIMP), and the test set comprised 115 projections (83 LM and 32 AIMP).

**Figure 2:**
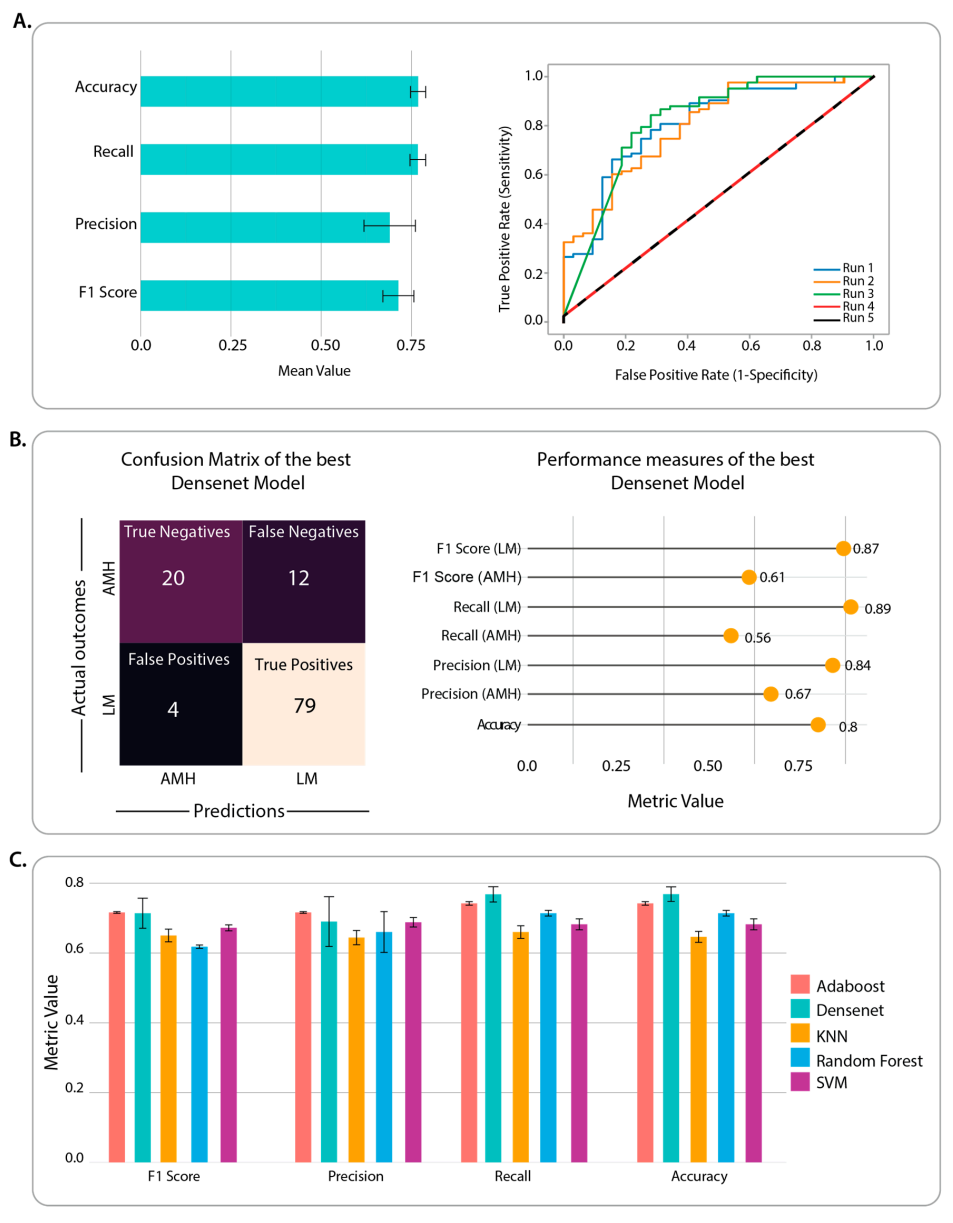
(**A**) The test-set performance of the DenseNet196 model over five runs of cross-validation is represented as bar plots and receiver operator characteristic (ROC) curves. The curves for Run 4 and Run 5 are identical and overlaid on top of each other. The bar plots represent the weighted average of the performance metrics (accuracy, recall, precision, and F1-score) across five runs. The error bar represents the standard error. (**B**) The confusion matrix representing the details of predictions made by the best-performing DensNet196 model (Run 1) and performance metrics in predicting LM and AIMP projections in the corresponding test set (20% of held-out data in Run 1 of 5-fold cross-validation). (**C**) The comparison of the DenseNet196 classifier with the traditional machine learning algorithms (AdaBoost, k-nearest neighbour (KNN), Random Forest, and Support Vector Machine (SVM)); the bar plots represent the weighted average of the performance metrics (accuracy, recall, precision, and F1-score) across five runs. The error bar represents the standard error.

Among selected CNN architectures pre-trained on the ImageNet dataset, DenseNet169 achieved the highest predictive power on the validation set (validation accuracy = 0.84). The predictive power of DenseNet169 was assessed on the test set (115 unseen images) using multiple metrics (Figure 2A). The class-specific precision and recall were averaged with the consideration of the class imbalance (i.e., weighted average). The best-performing DenseNet169 model was achieved via the first run of cross-validation (c.f. Run 1 in Figure 2B, ROC curves) with an accuracy of 0.80 on the test set (Figure 2B). The test accuracy of DenseNet169 as a standalone feature learning and classifier was higher than traditional classifiers (Figure 2C). However, the performance improvement was only significantly higher as compared to SVM and KNN (p-value < 0.05, paired, two-tailed t-test). Since DenseNet169 performed better or on par with the other classifiers, it was used for the subsequent patient-level prediction interpretation. All the analyses were performed on the Google Collaboratory platform’s GPU instance with 12.7 GB RAM and 78.2 GB disk space.

### 3.2. Identification of Classification Features and Examination of Misclassified Images

**Figure 3:**
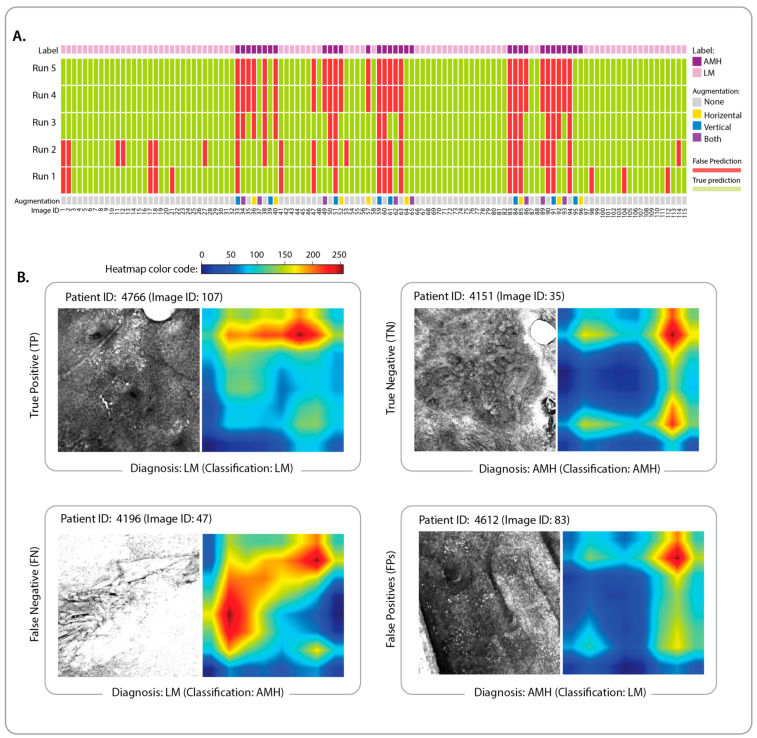
(**A**) Patient-level predictions of LM and AIMP images in the test set across five runs of the cross-validation. The heatmap represents the false predictions (false positives and false negatives) in red and the correct predictions (true positives and true negatives) in light green. Each 2D projection image (equivalent to an RCM stack is identified by a unique ID (Supplementary Table S1) and colour-coded based on the diagnosis (LM or AIMP) and augmentation of the 2D projections (vertical flip, horizontal flip, both, and none, i.e., no augmentation). (**B**) Selected projections in the test set and their corresponding Grad-CAM heatmaps enabling the interpretation of false and true predictions of LM (positive) and AIMP (negative) diagnoses. The colour code is identical for all heatmaps.

We examined predictions made by DenseNet169 models for each of the 115 projected images in the test set across five runs of cross-validation (Figure 3A). Image IDs in this figure can be mapped to the corresponding RCM stacks using Supplementary Table S3. To further understand factors contributing to the model’s false or true predictions, we plotted Grad-CAM heatmaps of selected images (Figure 3B) from the test set. The selection criteria were to include examples of LM and AIMP patients that are correctly classified (i.e., a true positive and a true negative) as well as examples of incorrectly diagnosed images (i.e., a false positive and a false negative) across the majority of the runs. We limited the selection to non-augmented images. The Grad-CAM heatmaps of the remaining test images are available in the GitHub repository (see Code Availability).

### 3.3. Impact of the Use of Projection in the Classification Pipeline

**Figure 4:**
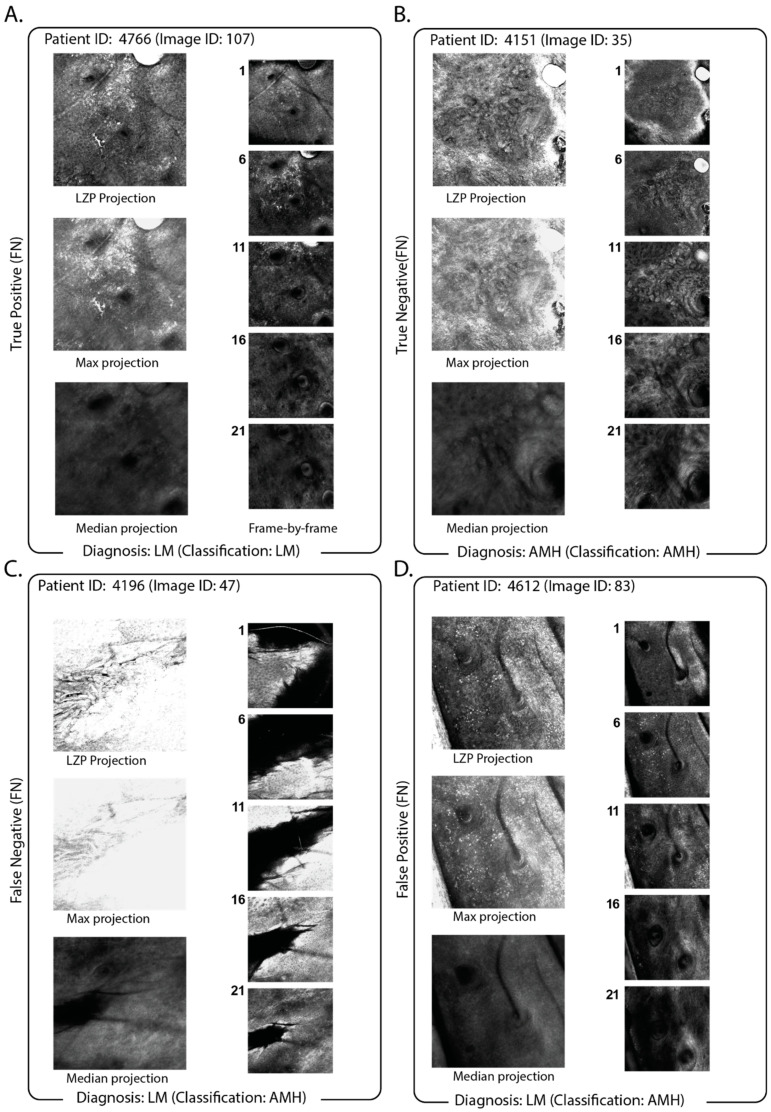
Comparison of LZP projection vs. max- and median-projection for exemplary classification outcomes. Exemplary data for (**A**) LM-diagnosed image stack correctly classified at L; (**B**) AIMP-diagnosed image stack classified as AIMP; (**C**) LM-diagnosed image stack misclassified as AIMP; and (**D**) LM-diagnosed image stack misclassified as LM. For all panels, projections are shown on the left (LZP: top; max-projection: middle; median-projection bottom) with individual slices at specific depths (z = 1, 6, 11, 16, 21) shown inset on the right. Max-projection is generated by taking the maximum value pixel across all slices of the stack, and median-projection is generated by taking the median value pixel across all slices of the stack.

To examine the effect of the projection, we visually compared projections using LZP with slice-by-slice clinician diagnosis to examine how well LZP projection preserved diagnostic markers in our original RCM stacks. Representative images are shown for each class in Figure 4, alongside the maximum z-projection (the highest pixel intensity at each location) and the median z-projection (the median pixel intensity at each location). Figure 4A indicates a representative true positive, that is, an LM diagnosis classified as LM where the stack had atypical, enlarged melanocytes and dendritic cells present at superficial levels indicating pagetoid spread. This was preserved in the projection, indicating that melanocytes were present at most levels within the stack. Figure 4B shows a representative true negative, that is, an AIMP-diagnosis that is classified as AIMP. The stack showed diffuse enlarged melanocytes at the basal layer with no dendritic cells. In the projection, the air bubble artifact in the top right is preserved, though it did not interfere with the correct classification being made. Figure 4C shows a representative false positive, that is, an AIMP-diagnosis classified as LM. There, the stack had diffuse enlarged melanocytes at the basal layer, with no pagetoid spread and no dendritic cells. The melanocytes were retained by projection. However, the information regarding at which depth the melanocytes were located was removed during projection.

Lastly, Figure 4D shows a representative false positive, that is, an LM-diagnosis classified as AIMP. There, the stack was acquired too early in superficial skin layers, and the presence of a skin fold prevented the acquisition of the whole en-face image. A pagetoid spread of non-dendritic melanocytes was present; however, irregular skin surface and non-perpendicular z images made it difficult to interpret pagetoid spread.

LZP can be seen to outperform simple max-projection since the individual detail and diagnostic markers remain clear (e.g., Figure 4A, Figure 4B true positive and true negative, respectively). However, when there are frames that are saturated at maximum brightness, these can dominate the signal in the projection (Figure 4C), and where the image stack is bright in different regions, this local information is lost upon projection. Likewise, in Figure 4D, marker information that shows clearly enlarged melanocytes at the basal layer (Figure 4 inset) are potentially misinterpreted as being present in all slices of the stack when considering only the projection.

### 3.4. Comparison of Projection with Slice-by-Slice Classification

We compared the classification of projections to the classification of individual slices at the slice-by-slice level. We revisited all stacks to add clinician diagnosis to individual slices as containing LM features, AIMP features, or non-pathological skin layers, respectively, since not all slices in a stack contained pathology. This increased the total number of images (training set: 4692, test set: 379) but also altered the problem to a ternary classification problem (no pathology, LM, or AIMP). The best-performing model for this ternary classifier was SVM (with Resnet101 used for feature extraction) which achieved an average test accuracy of 0.59 (precision = 0.64, recall = 0.59, and F1-score = 0.61, weighted average).

## 4. Discussion

We optimised our model to deliver a binary classification that could differentiate between AIMP and LM samples with a test accuracy of 0.80. Our approach was robust in that we were agnostic to a particular architecture, trying a variety of approaches and testing which had the highest accuracy and AUC. For different pathologies or diseases, a similar agnostic approach could be applied to the dataset to identify the architecture best suited for efficient and accurate classification and diagnosis.

The utilisation of deep learning models for the analysis of RCM images has been on the rise, as evidenced by recent studies reviewed by Malciu et al. [32]. For instance, a modified pre-trained ResNet with a shallower depth has been developed to identify lentigos in RCM mosaics [16]. The InceptionV3 architecture combined with data augmentation and transfer learning was used by Zorgui et al. [33] for RCM-based lentigo diagnosis. Kaur et al. [34] proposed a hybrid deep learning approach that integrates unsupervised feature extraction with supervised neural network classification for skin lesion diagnosis using RCM images.

While the application of computer-aided systems in the diagnosis of skin lesions using digitised slides is still limited, deep learning and traditional ML models have been extensively evaluated for their effectiveness in diagnosing skin lesions using dermoscopy images, as reviewed by Kassem et al. [35]. These evaluations have led to the development of several skin lesion classifiers, including those that employ pre-trained convolutional neural network (CNN) models and custom CNN architectures, such as multi-ResNet ensembles [36], depth-wise separable residual convolutional networks [37], and CNNs with attention residual learning [38]. Alternative techniques, such as clustering using features learned from Levenberg–Marquardt neural networks and stacked autoencoders [39], denoising adversarial autoencoders, and deep learning feature extraction combined with traditional ML methods such as support vector machines (SVM), random forest (RF), and multi-layer perceptron (MLP) have also been explored [40].

Training data sets for previous RCM image analysis studies have included single images, sometimes pre-selected in the vicinity of the dermoepidermal junction (DEJ) [11], RCM mosaics [16], or 3D reconstructions [33]. In contrast, we utilised a projection approach to project 3D and volumetric image data into a 2D representation of that volume as our computational performance was significantly optimised since we could use compressed single JPG images instead of large raw multi-layer TIFF stacks, greatly reducing the memory overhead (projection~500 kB; stack~100 MB).

Projection is, of course, not without drawbacks. First, it requires good alignment between the individual slices of a stack, and it is influenced by any drift in x- and y- as the operator moves deeper into the tissues. Similarly, where individual slices are saturated or overly bright, this saturated signal may dominate in the final projection. An example of this is shown in Figure 4C, where saturation in individual slices is localised to specific regions, but upon projection, the entire image is saturated, in that instance resulting in misclassification. Improvements in performance may be achievable through auto-adjustment of contrast or exclusion of saturated slices prior to projection, and, similarly, drift correction through registration of the slices in x- and y- may improve classification accuracy and is computationally inexpensive [41]. Projection methods are ultimately implementations of dimensionality reduction to imaging data and thus require compromise in which information they preserve or sacrifice. LZP here has proven suitable for the accurate classification of melanoma subtypes, and recent updates from the same team include deep learning to optimise structure-specific projections, which may yield further increases in accuracy [18].

One alternative to projection is to run a slice-by-slice classifier. This requires a clinician to provide a slice-by-slice diagnosis at the slice level and necessitates a ternary rather than binary classifier since many slices contain no specific features for LM or AIMP. This is even more susceptible to class imbalance. Our attempts at ternary classification resulted in a significantly reduced diagnostic performance (accuracy = 0.59). A second possibility would be to classify data as a volumetric medical image, that is, as a 3D stack without any projection, such as by using a 3D CNN architecture [42]. In our analysis, we were computationally limited, and our attempts to read in all 3D stacks had a tenfold higher read time, exhausted RAM capacity, and even in simplified run conditions, training was not able to be completed within 10 h. While this problem could be solved with more GPUs and memory, we noted that 3D CNNs have a larger number of parameters compared to their 2D counterparts, which can also increase the risk of overfitting, especially when using a small sample size. Furthermore, the complex features in 3D images make it difficult to design convolutional kernels that can effectively capture these features [17], and visualising the features learned by 3D CNNs is more difficult than representing these features in a 2D format, such as through Grad-CAM heatmaps [43].

RCM-trained clinicians typically make their diagnosis while imaging through the disease tissue on a slice-by-slice basis. RCM is a non-invasive technique but can only image up to 300 µm in depth. Data from slices above and below the disease tissue can confound machine classification, especially when an artefact is present, such as an air bubble or a follicle, and this can become prominent in the final projection. Clinicians/technicians could adapt their imaging approach in order to derive more benefit from computer-aided diagnosis in the future by avoiding drift, not projecting past the pathology or imaging too early, and avoiding saturation in any slice of the overall stack (adjusting the exposure, laser intensity, or the imaging conditions to guard against this).

## 5. Conclusions

We implemented an accurate Densenet169 CNN architecture that could classify LM vs. AIMP with an accuracy of 0.80 on our test set of projected RCM image stacks. The most notable limitation of our study was the sample size, particularly in the AIMP dataset, and small sample sizes are prone to over-fitting. We mitigated this in our work by augmentation and transfer learning, but a larger dataset from a single operator in near-identical conditions would be optimal. These strategies could be complemented by computational approaches such as image generation by an adversarial generative network. Similarly, the diversity of the training cohort may not match the wider community, and the corresponding model may fail to give optimal results for communities with varying endogenous contrast agents, such as melanin, from the training dataset [44]. Better and broader datasets, perhaps incorporating clinical data to help develop multi-modal predictive models, may help to increase classification accuracy in future.

The AIMP definition is not currently in the World Health Organization (WHO) classification. Nevertheless, our pathologists reviewed all borderline cases, with the consensus being the best ground truth available to us at this time. Our study is retrospective in nature and deals only with cases from a single institution. An external validation set and comparison with multiple human RCM experts would enable the work to be expanded in scale, as well as consistent standards for AIMP definition and classification among professional organisations.

We demonstrated that machine learning algorithms could be used to provide an initial non-invasive classification between LM and AIMP which may help to classify which LM-like lesions can be safely monitored and which need immediate treatment. In other areas of medical imaging, ML-driven pre-selection of specific images has driven a reduction in diagnostic time, such as in the context of prostate cancer [10]. Further training of machine learning classifiers in other contexts, as well as training of operators in preparing ‘machine-friendly’ image stacks, will benefit patient outcomes in the field and the further implementation of computer diagnosis as technologies improve.

## Data Availability

The data and codes that support the findings of this study are openly available for non-commercial uses at a GitHub Repository: https://github.com/VafaeeLab/Dermatology-ML.

## Supplementary Materials

The following supporting information can be downloaded at: https://www.mdpi.com/article/10.3390/cancers15051428/s1, Table S1: Hyper-parameter settings used to train deep learning and machine learning models studied; Table S2: Details of images used for model training and validation; Table S3: Prediction outcomes of individual images across different runs of cross-validation.

## Abbreviations

LM	lentigo maligna
AIMP	Atypical Intraepidermal Melanocytic Proliferation
RCM	reflectance confocal microscopy
LZP	local z-projection
