# Supplementary material for: Computer-Aided Diagnosis of Melanoma Subtypes Using Reflectance Confocal Images
Source: Cancers (Basel). 2023 Feb 23;15(5):1428. doi: 10.3390/cancers15051428 (PMC10000703; doi:10.3390/cancers15051428)
Supplement: Supplementary file 1 [file cancers-15-01428-s001.zip › Supplementary Tables/Supplementary Table 1.pdf]

Supplementary Table 1

A. Hyper-parameter settings used to train deep learning models studied.

Deep Learning model	***Epochs***	***Batch Size***	***Optimizer***	***Lr***
DenseNet169	100	15	Adam	0.001
ResNet50	100	10	RMSProp	2e-5
ResNet101	100	15	Adam	0.01
6 Layer CNN	50	10	RMSProp	0.001
Inception V3	100	10	Adam	0.01
MLP	50	10	Adam	0.01
Vgg16	50	10	Adam	0.001

B. Hyper-parameter settings used to traditional machine learning models trained using features derived from ResNet101.

Feature selection method	***ML method***	***Parameters used***	***ROC_AUC***
***ResNet101** for Feature Vector (For the best results)*	**RF**	n_estimators=10, criterion='gini', max_depth=None, min_samples_split=2, min_samples_leaf=1, min_weight_fraction_leaf=0.0, max_features='auto', max_leaf_nodes=None, bootstrap=True, oob_score=False, n_jobs=1, random_state=None, verbose=0	AUC = 0.603
	**AdaBoost**	base_estimator=None, n_estimators=500, learning_rate=0.1	AUC = 0.681
	**SVM**	gamma='scale', class_weight='balanced',probability = True, kernel = ['linear', 'poly', 'rbf', 'sigmoid', 'poly']	AUC = 0.681
	**KNN**	n_neighbors = 5 metric = 'minkowski', p = 2	AUC = 0.611
